# Assessment of Electromagnetic Interference with Active Cardiovascular Implantable Electronic Devices (CIEDs) Caused by the Qi A13 Design Wireless Charging Board

**DOI:** 10.3390/ijerph120605886

**Published:** 2015-05-27

**Authors:** Tobias Seckler, Kai Jagielski, Dominik Stunder

**Affiliations:** The Research Center for Bioelectromagnetic Interaction (FEMU), Institute and Out-patient Clinic of Occupational Medicine, RWTH Aachen University, Pauwelsstr 30, 52074 Aachen, Germany; E-Mails: jagielski@femu.rwth-aachen.de (K.J.); stunder@femu.rwth-aachen.de (D.S.)

**Keywords:** intermediate frequency magnetic fields, pacemaker, defibrillator, electromagnetic interference, wireless charging, wireless power transfer, Qi A13 design, performance limits, EMF risk assessment

## Abstract

Electromagnetic interference is a concern for people wearing cardiovascular implantable electronic devices (CIEDs). The aim of this study was to assess the electromagnetic compatibility between CIEDs and the magnetic field of a common wireless charging technology. To do so the voltage induced in CIEDs by Qi A13 design magnetic fields were measured and compared with the performance limits set by ISO 14117. In order to carry this out a measuring circuit was developed which can be connected with unipolar or bipolar pacemaker leads. The measuring system was positioned at the four most common implantation sites in a torso phantom filled with physiological saline solution. The phantom was exposed by using Helmholtz coils from 5 µT to 27 µT with 111 kHz sine-bursts or by using a Qi A13 design wireless charging board (Qi-A13-Board) in two operating modes “power transfer” and “pinging”. With the Helmholtz coils the lowest magnetic flux density at which the performance limit was exceeded is 11 µT. With the Qi-A13-Board in power transfer mode 10.8% and in pinging mode 45.7% (2.2% at 10 cm distance) of the performance limit were reached at maximum. In neither of the scrutinized cases, did the voltage induced by the Qi-A13-Board exceed the performance limits.

## 1. Introduction

Wireless power transfer (WPT) is an increasingly used strategy to improve user convenience and mobility for electrical devices. WPT refers to a number of different technologies for transmitting power subdivided into non-radiative techniques using capacitive or inductive coupling, or radiative techniques such as microwaves or laser beam.

The WPT standard ‘Qi’ uses inductive coupling for power transfer from a base station to a mobile device. The transmitter coil in the base station generates a magnetic field which induces a voltage in the receiver coil of the mobile device. A new design within the Qi standard is the A13 application which has been developed for operation in the automotive environment to wirelessly charge devices, e.g., the battery of the drivers’ smartphone [1].

The investigated Qi A13 design wireless charging board (Qi-A13-Board) utilizes a linear array of three rectangular shaped coils (53 × 45 mm) placed below a touch electrode. The Qi-A13-Board operates at the intermediate frequency of 111 kHz in either an idle state or in the power transfer mode. In the idle state the board issues sequential pings on each of its three coils in order to detect if a receiver is present. This pinging mode can be activated by objects placed on the top of the capacitance-based touch electrode and terminates if no answer is received. Once a receiver has been recognized each of the coils is scanned to determine which coil is best coupled with the receiver. The selected coil is then energized for power transfer with a maximum delivered power of 5 W (power transfer mode). However, the actual level of the transferred power depends on the receiver (mobile device) requirements [1,2].

With the magnetic field emission due to the inductive coupling, there arises the question of the safety to humans with cardiovascular implantable electronic devices (CIED) as electromagnetic interference (EMI) can result in life-threatening situations [3,4,5]. Devices with sensing capabilities such as permanent pacemakers (PPMs) or implantable cardioverter-defibrillators (ICDs) are especially susceptible to electromagnetic fields in the intermediate frequency range [6,7], because of potential misinterpretation of induced voltages as cardiac signals. Safety considerations have become more and more important as the number of new implantations of PPMs and ICDs increases every year as an annual European survey shows [8,9,10,11]. In 61 countries worldwide, just in 2009, over 1.3 million PPMs and ICDs were implanted, mostly utilizing bipolar leads (in Europe and the USA more than 99%) [12].

Guidelines for the protection of humans exposed to electric, magnetic or electromagnetic fields (EMF) such as established by the International Commission on Non-Ionizing Radiation Protection (ICNIRP) [13,14] or by the International Committee on Electromagnetic Safety (ICES) [15,16] do not consider product performance of medical devices. Therefore compliance with these guidelines may not necessarily preclude interference and EMI with CIED may occur below the recommended reference levels [14]. Furthermore, a current review reasoned concerns about sufficiency of these guidelines to protect CIEDs from hazardous interference by WPT and therefore recommended that additional research is required [17].

Electromagnetic compatibility (EMC) of CIEDs is addressed by the international standard ISO 14117 [18]. This standard sets performance limits up to 3 GHz with the objective to prevent harm due to EMF, whether through malfunction, damage or heating of the device. Malfunctions from sensing EMF as cardiac signals require the lowest performance limits given by this standard. The limits rise linearly in the concerning frequency range (3 kHz to 167 kHz) and distinguish between unipolar and bipolar leads. To evaluate EMC performance three general test methodologies are specified: test signal one is a continuous sinusoidal wave with a frequency between 16.6 Hz and 1 kHz; test signal two is a modulated signal, carrier frequency, between 1 kHz and 150 kHz and switched to create 100 ms burst; test signal three is a modulated signal, carrier frequency, between 150 kHz and 10 MHz, with the carrier amplitude modulated with a 130 Hz sinusoidal wave and switched to create 100 ms burst. For the respective frequency of the Qi-A13-Board (111 kHz) the performance limits are 333 mV for CIEDs with unipolar leads and 33.3 mV for CIEDs with bipolar leads. It is important to understand that the performance limits can only demonstrate that CIEDs produce an appropriate level of EMC. However, for which exposure conditions the levels apply is not stated due to the unknown correlation between performance limits and strength of external EMFs.

The objective of this study was to conduct a risk assessment of CIED patients by investigating conditions in which the performance limits of CIEDs are fulfilled for magnetic fields at 111 kHz. We therefore measured the induced voltage in a torso phantom in consideration of the most EMI affecting parameters: type and position of the CIED lead as well as field distribution [19,20] in order to test under real case (inhomogeneous) and worst-case (homogeneous) exposure conditions. For a risk assessment we then compared the measured induced voltage with the performance limits.

## 2. Methods

The experimental setup consisted of either a set of Helmholtz coils or the Qi-A13-Board, a torso phantom and a self-developed measuring system. With the self-developed measuring system induced voltages were measured using standard endocardial pacing/sensing leads—one unipolar and one bipolar lead. The measuring system was exposed to homogenous fields emitted by the Helmholtz coils or inhomogeneous fields of the Qi-A13-Board in both operation modes (power transfer mode and pinging mode) at four different positions within the torso phantom. All measurements were done in a dedicated shielding chamber which attenuates external magnetic fields by 100 dB to 110 dB in the frequency range from 100 kHz to 1000 kHz.

### 2.1. Torso Phantom and Measuring System

**Figure 1:**
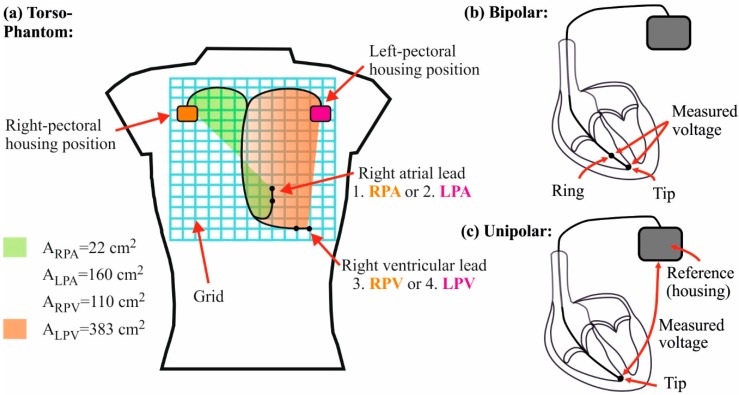
(**a**) Schematic diagram of the torso phantom. All implantation sites are indicated: 1. Housing: right-pectoral. Lead: atrium (RPA). 2. Housing: left-pectoral. Lead: atrium (LPA). 3. Housing: right-pectoral. Lead: ventricle (RPV). 4. Housing: left-pectoral. Lead: ventricle (LPV).The green area marks the unipolar induction area for the RPA implantation site (A_RPA_). The orange area marks the unipolar induction area for the LPV implantation site (A_LPV_). A_RPV_ and A_LPA_ are the unipolar induction areas of the implantation sites RPV and LPA. (**b**) Sensing mechanism of a bipolar lead. A differential (the housing is the reference electrode) voltage is measured between tip and ring electrode. (**c**) Sensing mechanism of a unipolar lead. A voltage is measured between tip and the reference (housing) electrode.

A schematic diagram of the torso phantom containing the measuring system is shown in Figure 1. The human-shaped torso resembles a male upper body of size M and has a total volume of 30 L. The torso was filled with deionized water and 0.14% sodium chloride to obtain a solution with an electric conductivity of 0.25 S/m which simulates in average the electrical properties of a human body (*cf.* 0.2 S/m [21] and 0.3 S/m [22]). In order to assure the measuring system’s position and thereby define the implantation sites in the torso phantom, the implant-housing and the lead were attached to a grid (27 × 30 cm). Two implant-housing positions (left- and right-pectoral) and two lead positions (ventricle and atrium) were used to scrutinize the influence of the implantation site on the induced voltage (Figure 1). All materials selected for the torso phantom and the grid are made of plastic and can therefore be considered to have a relative permeability of 1 to ensure that they do not affect the distribution of the magnetic field.

The induced voltage was measured by a self-developed circuit mounted in an implant-housing. Therefore it is possible to connect the Setrox S53 bipolar lead (length: 53 cm, Biotronik, Berlin, Germany) and the Capsure Sense 4073 unipolar lead (length: 58 cm, Medtronic, Minneapolis, MN, USA). The circuit in the implant-housing consisted of a differential amplifier and an optical output to transmit the induced voltage signal via an optical fiber (length 5 m) to a receiver circuit. The differential amplifier worked linearly for input signals between ±150 mV at 111 kHz and amplified the input signal by a factor of 10. The measured voltage signal is digitalized after the receiver circuit with a 9223 analog input device (National Instruments™, Austin, TX, USA) with differential input channels (±10V), 1 MHz sampling rate and 16 bit resolution. The described measurement system was therefore capable of measuring the induced voltage in a bipolar as well as in a unipolar lead.

### 2.2. Homogenous Field Exposure by Using Helmholtz Coils

The homogenous magnetic field was generated by a set of Helmholtz coils with a diameter of 0.71 m, which were driven by two 7224 amplifiers (1.1 kW max, DC-300 kHz, AE TECHRON^®^, Elkhart, IN, USA). The produced magnetic field was homogeneous along the rotation axis (±5% between the coils) and in the symmetry plane between the coils (±3% within ±18 cm from the center). The coils were therefore capable of exposing the whole volume around the grid in the torso phantom with a homogeneous magnetic field.

The magnetic flux density was controlled by measuring the current in a series shunt (12 Ω). Each of the coils had two windings and an inductance of 6 µH. A magnetic flux density of 5.1 μT/A was generated. The maximum magnetic flux density for sine bursts at 111 kHz was 30 µT.

The maximum exposure was set to 27 µT which is the ICNIRP reference level from 2010 for general public exposure to time varying magnetic fields at 111 kHz [14]. The reference level of the respective ICNIRP guideline from 1998 for general public exposure is 6.25 µT [13]. ICNIRP 2010 only replaces the ICNIRP 1998 exposure limits in the frequency range of 1 Hz to 100 kHz. However values are provided up to 10 MHz for some guidance. In order to comply with both guidelines the torso phantom was exposed homogenously to magnetic fields (111 kHz sine-bursts of 100 ms duration) from 5 µT to 27 µT (in 1 µT steps).

For two measurements with the unipolar lead the magnetic flux density range was changed because the induced voltage exceeded the linear input range (±150 mV) of the differential amplifier of the measurement system. For the left-pectoral ventricle position (LPV) the magnetic flux density range was set from 1 µT to 8 µT and for the left-pectoral atrium position (LPA) it was set from 1 µT to 9 µT.

### 2.3. Inhomogeneous Field Exposure by Using the Qi-A13-Board

The measurements of the voltage induced by the Qi-A13-Board were done in two operating modes (power transfer and pinging mode). In the power transfer mode an AVID-Receiver Qi Receiver Simulator from AVID Technologies (Burlington, MA, USA) was placed on the Qi-A13-Board. The AVID-Receiver ensures continuous power transfer with the maximum deliverable power of 5 W by connecting external load of 5 Ohm. The position of the AVID-Receiver on the Qi-A13-Board and the position in relation to the torso phantom of both devices together, were then systematically changed to find the position where the induced voltage has the highest amplitude (worst-case position). This was done for all four implantation sites and each time the induced worst-case voltage was recorded.

The procedure of identifying the worst-case position was repeated with the Qi-A13-Board in the pinging mode—that means neither the AVID-Receiver nor any other receiver was used. The pinging mode was activated by touching the Qi-A13-Board briefly with one hand. Again for LPV-, LPA-, RPV- and RPA-position, the induced worst-case voltages were measured. In this mode a second measurement of the induced voltages was done at 10 cm distance to the previously identified worst-case position. A 10 cm distance was considered to assess the attenuation of the induced voltage. The distance was created along a line that was perpendicular to the surface of the torso phantom.

To determine the field distribution of the Qi-A13-Board in both operating modes, magnetic flux density measurements were performed with a calibrated Exposure Level Tester 400 (ELT-400) from Narda Safety Test Solutions^®^ (Pfullingen, Germany). Unless specified differently, the measurements were averaged over one second and taken in the Root-Mean-Squared (RMS) mode. The field strength mode was set to 320 µT (range low) and the selected frequency range was set from 30 Hz to 400 kHz. The measurement uncertainty in the range from 50 Hz to 120 kHz is declared not to be higher than ±6% according to the ELT-400 manual.

As a standard approach [23], we first conducted field measurements with a 100 cm^2^ isotropic magnetic field probe. For the measurements, the position of the probe was slewed systematically over the Qi-A13-Board to find the spot where the magnetic flux density reaches the highest amplitude in both operation modes. In the power transfer mode the AVID-Receiver also was moved on the board in order to find the worst-case magnetic flux density.

The 100 cm^2^ probe’s diameter of around 12 cm allowed only a rough determination of the field distribution of the Qi-A13-Board. Therefore we conducted a second measurement using an isotropic magnetic field probe with a small effective area of 3 cm² and an outer diameter of 3 cm. This allowed measurements of highly inhomogeneous magnetic fields and even small recesses of the Qi-A13-Board to be assessed.

The field measurements were conducted systematically on a grid with the dimension of 350 × 300 mm in two planes parallel to the coil array of the Qi-A13-Board at distances of 2 cm and 10 cm. The measuring points in those planes were located on the grid with a spacing of 3 cm to 4 cm. Some points had to be slightly shifted or excluded due to protrusions of the Qi-A13-Board or the AVID-Receiver (in case of power transfer mode). The measuring points refer to the tip of the probe and a distance correction to the center of the probe is taken into account.

In power transfer mode the AVID-Receiver on the Qi-A13-Board was positioned so that the magnetic flux density reached the highest amplitude but the power transfer was still stable during the entire measurement. In pinging mode the ELT-400 was set to MAX HOLD to ensure measurement of the maximum magnetic field. All field measurements were assessed in time and frequency domain.

### 2.4. Signal Analysis

The signal analysis was performed using MATLAB^®^ Release 2014b from The MathWorks, Inc. (Natick, MA, USA). For the estimation of the amplitude of the induced voltage (sine-burst signals) the least squares method was applied for every M = 10 consecutive samples using Equation (1) as curve fitting function:

(1) $${\hat{y}}_{\text{k}}=c_{1,\text{k}}\sin\left(\omega t\right)+c_{2,\text{k}}\cos(\omega t)$$

The 10 samples of the measured data ($y_{\text{m}}$) include the information of one period of the 111 kHz sine wave (sample rate 1 MHz). With this method N=10,000 amplitude estimations were conducted during one sine-burst with the duration of 100 ms. The amplitude $(c_{\text{k}}$) was calculated with the estimated parameters using Equation (2):

(2) $$c_{\text{k}}=\sqrt{{c_{1,\text{k}}}^{2}+{c_{2,\text{k}}}^{2}}$$

The arithmetic mean of the estimated amplitudes was then determined using Equation (3) to obtain the induced voltage $u_{i}$:

(3) $$u_{i}=\frac{1}{N}\overset{N}{\underset{\text{k}=1}{\sum }}c_{\text{k}}$$

Additionally, the coefficient of determination was calculated using Equations (4) and (5) to examine the quality of each sine-fit:

(4) $${r_{k}}^{2}=1-\frac{\sum_{\text{m}=1}^{M}{\left(y_{\text{m}}-{\hat{y}}_{\text{k},\text{m}}\right)}^{2}}{\sum_{\text{m}=1}^{M}{\left(y_{\text{m}}-{\bar{y}}_{k}\right)}^{2}}$$

(5) $$r^{2}=\text{min}({r_{k}}^{2})$$

As a second quality criterion the relative standard deviation (STD) of the estimated amplitudes $c_{\text{k}}$ were calculated. In order to estimate the induced voltage for the not tested magnetic flux densities the results of $u_{i}$ were fitted with the linear function in Equation (6):

(6) $$u_{i,extrapolation}=a_{1}x+a_{2}$$

## 3. Results

### 3.1. Homogenous Field Exposure by Using Helmholtz Coils

**Figure 2:**
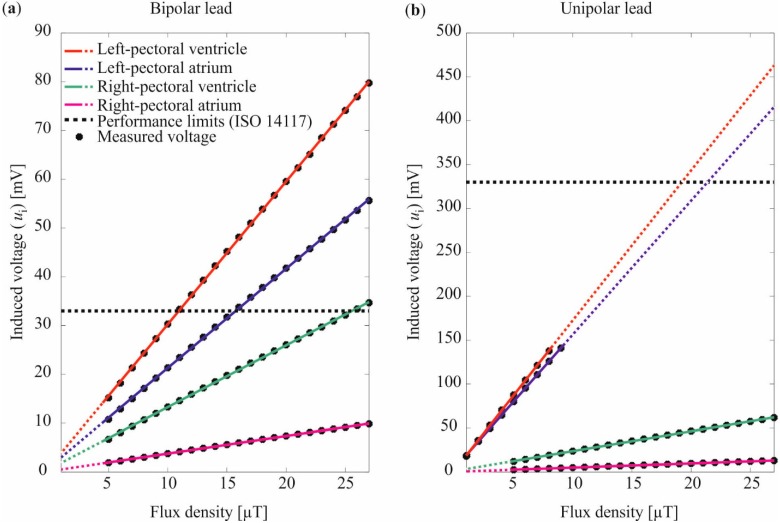
Results of the induced voltage for the homogenous exposure for (**a**) the bipolar lead, and (**b**) the unipolar lead. The linear fit to the measured values is indicated by the solid lines. The dashed lines indicate the extrapolation of the linear fit to the ranges where no induced voltages were measured due to the operation range of the used differential amplifier of the measurement system.

The results of the induced voltage for the four implantation sites and the two lead types are shown in Figure 2 together with the performance limits of ISO 14117. The results of the linear fit of the measured voltage are given in Table 1 and shown in Figure 2. The induced voltage and the performance limit are always expressed as peak values, however, the magnetic flux density as root mean square.

As shown in Table 1 and Figure 2 the induced voltage was strongly dependent on the implantation site and the lead type (bipolar/unipolar). On the left-pectoral implantation sites the voltages induced in unipolar leads were 5.8 to 7.6 times higher in comparison to the bipolar leads (*cf.* a_1_ Table 1). However, the performance limit for bipolar leads is 10 times lower than for the unipolar leads [18]. Thus, the performance limit is exceeded at lower magnetic flux densities (*cf.* Figure 2). The lowest magnetic flux density at which the bipolar performance limit (33.3 mV) was exceeded is 11.0 µT. The lowest magnetic flux density at which the unipolar performance limit (333 mV) was exceeded is 19.4 µT considering linear correlation between the flux density and the induced voltage (*cf.* Figure 2 dashed lines). In both cases the first exceedance occurred at the left-pectoral ventricle position (red curve in Figure 2).

**Table 1:** Results for the linear fit of the induced voltage.

Lead Unipolar/Bipolar	Housing Position	Lead Position	$a_{1}$ (V/mV) Equation (6)	$a_{2}$ (mV) Equation (6)	$r^{2}$ of the Linear Fit
Bipolar	Left-pectoral	Ventricle	2.93	0.96	0.9999
		Atrium	2.04	0.95	0.9998
	Right-pectoral	Ventricle	1.27	0.60	0.9998
		Atrium	0.36	0.15	0.9998
Unipolar	Left-pectoral	Ventricle	17.09	1.68	0.9999
		Atrium	15.55	1.68	0.9999
	Right-pectoral	Ventricle	2.26	1.09	0.9997
		Atrium	0.48	0.20	0.9998

On the right-pectoral implantation sites, clearly less voltage was induced than on the left pectoral implantation sites—2.3 to 5.7 times lower for the bipolar lead and 7.6 to 32.4 times lower for the unipolar lead (*cf.* $a_{1}$ Table 1). The voltage induced in the unipolar lead was higher than in the bipolar lead like it was on the left-pectoral implantation sites. Only the right-pectoral ventricle position exceeded the performance limit at 25.6 µT (*cf.* green curve Figure 2a). The other three implantation sites (RPA unipolar and bipolar, RPV unipolar) kept clearly below the limits of ISO 14117 even at maximum exposure. The quality criterion $r^{2}$ states a very good compliance of the measured voltage with the linear functions (*cf.* Figure 2 compliance of black dots with colored lines).

### 3.2. Inhomogeneous Field Distribution by Using Qi-A13-Board

**Figure 3:**
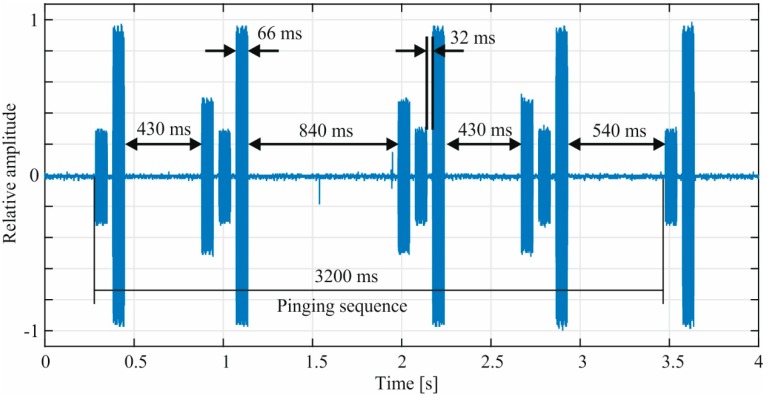
Pinging sequence recorded during pinging mode of the Qi-A13-Board. The amplitude is normalized to one.

Figure 3 shows a typical pinging sequence recorded during pinging mode of the Qi-A13-Board. The pinging sequence had a length of 3200 ms and consisted of two or three consecutive sine-bursts with a frequency of 111 kHz. The bursts had a width of 66 ms and a 32 ms pause in between. The pause between the consecutive bursts varied from 430 ms to 840 ms during our recordings. The burst amplitudes varied depending on which of the three coils on the Qi-A13-Board was driven. For the evaluation of the induced voltage (*cf.* Section 3.3) always the burst with the highest amplitude was chosen.

As described before, the pinging mode can be activated by objects placed on the top of the capacitance-based touch electrode and terminates if no answer is received. During the measurements we noticed, that the pinging mode stayed activated as long as the capacitance-based electrode was touched. In this case the pinging sequence (*cf.* Figure 3) was repeated by the Qi-A13-Board.

**Figure 4:**
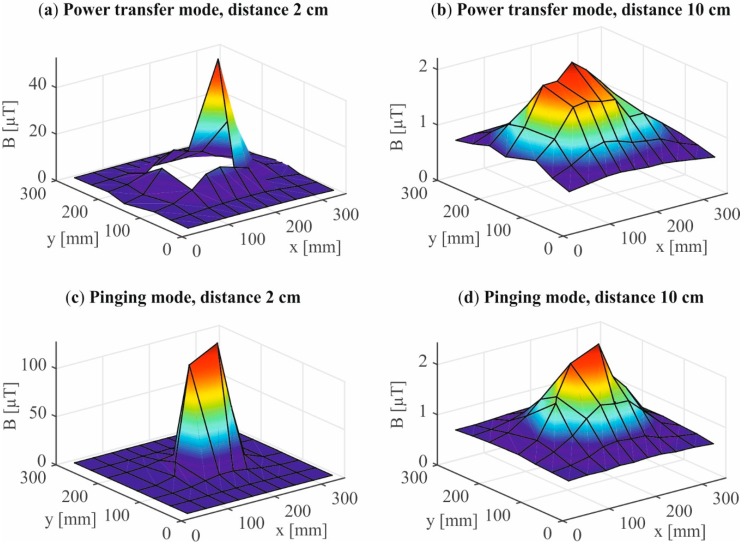
The measured magnetic flux density *vs.* the coordinates x and y on the plane is shown for the power transfer mode at two distances (2 cm (**a**) and 10 cm (**b**)) and for the pinging mode at two distances (2 cm (**c**) and 10 cm (**d**)). The missing values in the plot of the power transfer mode at 2 cm (**a**) are caused by the housing of the AVID-Receiver.

In Figure 4 the inhomogeneous field distribution of the Qi-A13-Board is depicted for the power transfer (a, b) and pinging mode (c, d). It was determined using the 3 cm^2^ magnetic field probe. The highest magnetic flux density (127 µT) occurred during the pinging mode at a distance of 2 cm over the center of the Qi-A13-Board (Figure 4c). At a 10 cm distance the magnetic flux density drops about 98% to 2.4 µT at maximum (Figure 4d). The maximum value measured in the power transfer mode is 53 µT at 2 cm distance and alongside the AVID-Receiver (Figure 4a). At a 10 cm distance the magnetic flux density drops about 96% to 2.2 µT (Figure 4b).

The maximum magnetic flux density measured with the 100 cm² probe was 12.5 µT in the pinging mode and 4.7 µT in the power transfer mode. The background noise level was quantified in the measured spectrum (30 Hz to 400 kHz) to a maximum value of 0.65 µT for all measurements on the Qi-A13-Board.

### 3.3. Inhomogeneous Field Exposure by Using the Qi-A13-Board in Power Transfer Mode

**Figure 5:**
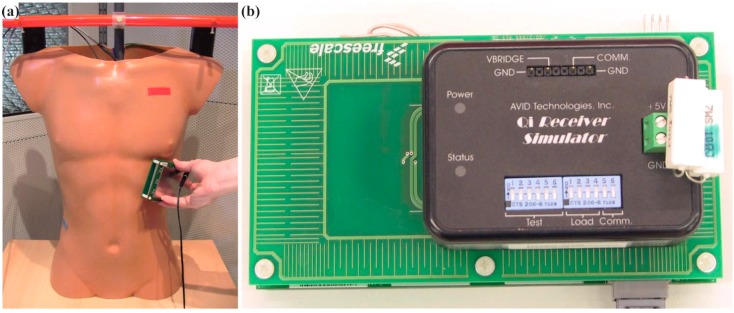
(**a**) Torso phantom and Qi-A13-Board (pinging mode) on the lateral wall; (**b**) AVID-Receiver close to the edge of the Qi-A13-Board. If the AVID-Receiver was moved further over the edge the connection was interrupted.

In the power transfer mode 10.8% of the performance limit was reached at maximum (*cf.* Table 2 LPA unipolar lead). Using a bipolar lead 7.5% of the performance limit was reached at maximum (RPA). The induced voltage in both implant-housing positions and with both lead types was higher at the atrium position than at the ventricle positions—20% to 67% for the bipolar lead and 60% to 88% for the unipolar lead. In Table 2 the induced worst-case voltages that occurred during the exposure with the Qi-A13-Board in power transfer mode are given. The highest voltages were induced when the AVID-Receiver and the Qi-A13-Board together were in the worst-case position. The AVID-Receiver was then close to the edge of the Qi-A13-Board (*cf.* Figure 5b). The worst-case position of the AVID-Receiver and the Qi-A13-Board on the torso phantom changed whenever the implantation site or the lead type was changed. In general it can be stated, that the worst-case position was on the lateral wall of the phantom at a height slightly above the tip of the lead (*cf.* Figure 5a).

**Table 2:** The worst-case voltage induced by the exposure with the Qi-A13-Board in power transfer mode.

Lead Type	Housing Position	Lead Position	$u_{i}$ (mV)	Percentage of the Performance Limit (%)	Relative STD (%)	$r^{2}$ Equation (5)
Bipolar	Left-pectoral	Ventricle	2.0	6.0	2.3	0.9994
		Atrium	2.4	7.2	1.3	0.9997
	Right-pectoral	Ventricle	1.5	4.5	1.3	0.9992
		Atrium	2.5	7.5	1.4	0.9997
Unipolar	Left-pectoral	Ventricle	19.1	5.7	0.1	>0.9999
		Atrium	35.9	10.8	0.1	>0.9999
	Right-pectoral	Ventricle	11.1	3.3	0.1	0.9999
		Atrium	17.8	5.3	0.3	0.9998

### 3.4. Inhomogeneous Field Exposure by Using the Qi-A13-Board in Pinging Mode

In the pinging mode 45.7% of the performance limit was reached at maximum (*cf.* Table 3 LPA unipolar lead). Using a bipolar lead 37.6% of the performance limit was reached at maximum (RPA). By increasing the distance between the Qi-A13-Board and the worst-case position on the torso phantom from 0 cm to 10 cm the induced voltage was reduced by 93% on average. This complies with the results of magnetic field measurements where the magnetic flux density drops about 98% at a distance of 10 cm (*cf.* Section 3.2.). Furthermore, at the left-pectoral implantation sites a higher voltage was induced than at the right-pectoral implantation sites for unipolar leads. Likewise for bipolar leads at the left-pectoral ventricle position a higher voltage was induced than at the right-pectoral ventricle position. Though, the induced voltage at the left-pectoral atrium position was lower than at the right-pectoral atrium position. In Table 3 the results of the induced voltage when exposed with the Qi-A13-Board in pinging mode at a distance to the phantom of 0 cm (worst-case) and 10 cm are given.

**Table 3:** Induced voltage at 0 cm distance (worst-case) and at 10 cm distance by the Qi-A13-Board in pinging mode.

Lead Type/Setup	Housing Position	Lead Position	$u_{i}$ (mV)	Percentage of the Performance Limit (%)	Relative STD (%)	$r^{2}$ Equation (5)
**Bipolar/0 cm distance**	Left-pectoral	Ventricle	6.72	20.4	0.2	0.9999
		Atrium	6.84	20.7	2.8	0.9999
	Right-pectoral	Ventricle	6.37	19.3	0.2	0.9999
		Atrium	12.40	37.6	0.1	0.9999
**Bipolar/10 cm distance**	Left-pectoral	Ventricle	0.72	2.2	2.0	0.9970
		Atrium	0.64	1.9	2.2	0.9952
	Right-pectoral	Ventricle	0.41	1.2	3.5	0.9873
		Atrium	0.52	1.6	2.7	0.9925
**Unipolar/0 cm distance**	Left-pectoral	Ventricle	54.12	16.4	0.1	>0.9999
		Atrium	150.72	45.7	0.1	>0.9999
	Right-pectoral	Ventricle	42.94	13.0	0.2	>0.9999
		Atrium	85.38	25.9	0.1	>0.9999
**Unipolar/10 cm distance**	Left-pectoral	Ventricle	5.31	1.6	0.3	0.9999
		Atrium	6.56	2.0	0.3	0.9999
	Right-pectoral	Ventricle	2.49	0.8	0.6	0.9996
		Atrium	3.37	1.0	0.5	0.9998

The worst-case positions for this operating mode were similar to the worst-case positions found for the power transfer mode. Hence, the worst-case positions still belongs to the lateral wall of the phantom model (*cf.* Figure 5).

The quality of the amplitude estimations for all shown measurements is very high (*cf.* $r^{2}$ Table 2 and Table 3). The lowest $r^{2}$ (0.9873) occurred at very low input voltage (*cf.* $r^{2}$ Table 3). Still this estimation explains 98.7% of the variation of the measured signal. For the results with higher induced voltages the $r^{2}$ value increases even more (>0.9999) due to the improving signal to noise ratio. The second quality criterion, the relative standard deviation, confirms the values of the amplitudes as well. The variation of the estimation was not higher than 3.5% and occurred in the same measurement as the lowest $r^{2}$. With increasing signal to noise ratio the relative STD reaches even values of 0.1% (*cf.* Table 2 and Table 3). The amplitude estimation function (Equation (1)) is therefore a precise measure for the induced voltage amplitude.

## 4. Discussion and Conclusions

### 4.1. Risk Assessment of CIEDs When Exposed to Magnetic Fields at 111 kHz

At homogenous exposure to 111 kHz magnetic fields the induced voltage exceeded the performance limits defined in ISO 14117 and therefore EMI with CIED is conceivable. The lowest magnetic flux density at which the performance limit was exceeded was 11 µT (*cf.* Section 3.1). This is about 77% above 6.25 µT which is the respective ICNIRP reference level for general public exposure at 111 kHz [13]. The situation needs to be reassessed as ICNIRP will soon announce the new guideline on limiting exposure to EMF in the range of 100 kHz to 300 GHz [24]. The exceedance of ISO 14117, however, cannot be equated with a hazard for patients with CIEDs. The hazard limit depends not only on the induced voltage and the frequency but also on the shape of the induced signal as well as construction features (e.g. filter design) of the CIED [25]. Therefore the hazard limit is individually different and might be higher than the performance limit.

According to Figure 2 the exceedance of the performance limits occurred at LPV-, LPA- and RPA-position for the bipolar lead and LPV- and LPA-position for the unipolar lead. However, this result should be understood in the context of the test conditions of ISO 14117. Devices programmed to unipolar sensing mode ought to be tested in 2.0 mV sensitivity setting and devices in bipolar sensing mode ought to be tested in 0.3 mV sensitivity setting. This means, if for the unipolar lead the same sensitivity setting and thus the same performance limit than for the bipolar lead had been considered, the induced voltage would be exceeded already at 1.83 µT (LPV-position in Figure 2b). These findings that EMI with CIEDs occurs at lower field levels when using unipolar instead of bipolar leads complies with *in vivo* investigations by Tiikkaja *et al.* [26]. They exposed 24 CIED patients with magnetic fields of different patterns (sine, pulse, ramp, and square waveform) with flux densities up to 0.3 mT. Only CIEDs tested in unipolar sensing mode were affected by the magnetic fields. Bipolar settings caused no interference.

The first exceedance of the performance limit under homogeneous field exposure at 11 µT is lower than the highest magnetic flux density measured at the Qi-A13-Board (127 µT with the 3 cm^2^ probe and 12.5 µT with the 100 cm^2^ probe, *cf.* Section 3.2). However, with the Qi-A13-Board not more than 45.7% of the performance limit could be reached. Thus, an EMI risk assessment of CIEDs exposed by the Qi-A13-Board cannot be performed on the basis of the results of the homogeneous exposure, because the risk would be overestimated.

**Figure 6:**
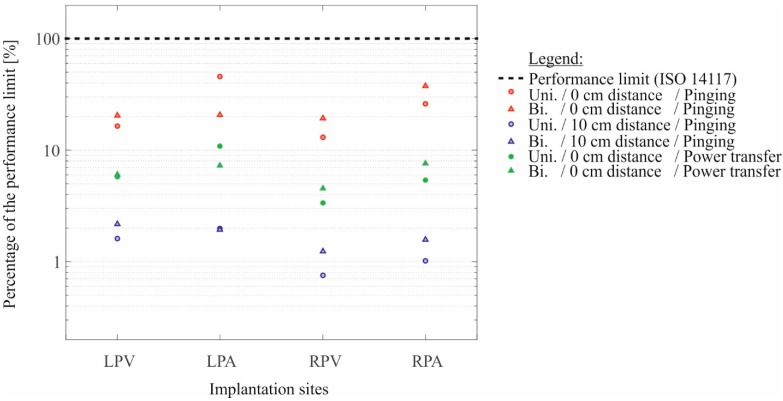
The percentage portion of the performance limits of ISO 14117 contextualized with the most EMI affecting parameters: the four implantations sites, the two lead types and the two operating modes of the Qi-A13-Board. Additionally the attenuation of the induced voltage at 10 cm distance is presented (compare red and blue markers).

For the inhomogeneous fields emitted by wireless power transfer base stations, e.g., Qi-A13-Boards so far no recommendations considering CIED patients have been published—in fact research needs have been addressed by Christ *et al*. [17]. In Figure 6 the induced voltages from the exposure with the Qi-A13-Board are summed up for all four implantations sites (LPV, LPA, RPV and RPA), for the unipolar and bipolar lead type as well as for power transfer and pinging mode. The results are shown in relation to the performance limit of ISO 14117 as the percentage portion of these limits. In neither of the scrutinized cases, the induced voltage exceeded the performance limits of ISO 14117. However, 45.7% of the performance limit was reached using the unipolar lead and 37.6% were reached using the bipolar lead at maximum (*cf.* Figure 6). The maxima of the induced voltage occurred when the Qi-A13-Board operated in the pinging mode which is in accordance with the field measurements where the highest magnetic flux density (127 µT) was also found in the pinging mode (*cf.* Section 3.2). At a distance of 10 cm between the Qi-A13-board and the torso phantom only a maximum of 2.2% of the performance limit was reached.

Regarding the influence of the implantation site at inhomogeneous field exposure: the left-pectoral position as well as at the atrium position generally a higher voltage was induced than at the right-pectoral position and the ventricle position (*cf.* Section 3.3 and Section 3.4). At the atrium position the induced voltage is about a factor of approx. 2 closer to the performance limit than it is at the ventricle position (*cf.* Figure 6). Thus, for inhomogeneous field exposure EMI with CIEDs is more likely to occur in the atrial lead.

Our results revealed that inhomogeneous exposure with Qi-A13-Boards does not exceed 46% of the performance limit even under worst-case conditions in a torso phantom. However, in consideration of general limitations of phantom models—torso shape, lead position and time variant tissue conductivity might be different in humans—a safety margin is recommended for transferring the results to humans with CIEDs. In our torso phantom a 10 cm distance of the Qi-A13-Board assured a safety margin where the induced voltage is nearly 50 times smaller than the performance limit.

Our method of measuring the induced voltage and comparison with the performance limits of ISO 14117 is a way of risk assessment which enables a general statement about potential EMI with CIEDs because all manufacturers are well-advised to test their devices according to the product standard to benefit from the presumption of conformity given by harmonized standards. In most other studies only a limited number of CIEDs were investigated which often limits their outcomes to the tested devices [7,27,28,29].

### 4.2. Correlation between Induced Voltage and Strength of EMF

Correlation of CIED’s interference input voltage with external EMF is a very complex matter. The induced voltages depend on so-called coupling factors that vary between extremely low and radio frequency fields. It also depends on the field distribution as our results of homogeneous and inhomogeneous field exposure confirm. The inhomogeneous field distribution is defined by the design, the geometry and the materials used of the field source which is in case of the Qi-A13-Board one of the three coils. To determine the induced voltage in previous studies phantom models [6,7,22,27,29,30,31,32,33], theoretical considerations [20,31,32,34] or numerical simulations [35,36] are used with different approaches for specific exposure conditions.

For unipolar leads exposed to homogeneous fields correlations between the external magnetic fields and induced voltage in the intermediate frequency range can be found in [20,30,31,32,33,34]. The correlations introduced are all based on Faraday’s law of induction (Equation (7)):

(7) $$u_{\text{uni}}\;\sim \;\omega BA$$

The A stands for the effective unipolar induction area (*cf.* Figure 1) and $u_{\text{uni}}$ is the induced voltage in a unipolar lead. The applicability of the induction law was stated to frequencies up to 50 kHz [30,31,32,33,34]. Only Irnich [20] indicated the applicability up to 1 MHz. However, this statement is only based on analytical results and no measurements are provided. In our study the dependency of the unipolar induction area is clearly shown (*cf.* Figure 2b).

For bipolar leads correlations were developed by Hille *et al.* [31,32], Irnich [20] and Mattei *et al.* [33]. Hille *et al.* [31,32] measured the voltage induced in a bipolar lead with a phantom model and Helmholtz coils for homogenous exposure. They found an interception point of the induced unipolar and bipolar voltage at around 9 kHz (investigated frequency range: 50 Hz to approx. 20 kHz). For frequencies higher than 9 kHz the voltage induced in the scrutinized bipolar lead was higher than in the unipolar lead. However, the presented measurement system had no optical fiber in order to prevent noise coupling into the connection cable. In addition the phantom used was not human shaped and the scrutinized bipolar lead had a distance of 15 mm between tip and ring electrode. In our study we used a bipolar lead with 10 mm distance between tip and ring electrode. According to Irnich [20] the tip-ring-distance has major influence on the induced voltage.

Irnich [20] derived a correlation analytically by comparing the coupling mechanism of unipolar and bipolar leads in magnetic fields. For a given magnetic flux density, he stated, that the voltage induced in a bipolar lead ($u_{\text{bi}}$) is reduced by a factor of d/l (l is the length of the lead and d is the tip-to-ring distance, *cf.* Equation (8)) compared to the voltage induced in a unipolar lead ($u_{\text{uni}}$):

(8) $$\frac{d}{l}=\frac{u_{uni}}{u_{bi}}\;\;\;\;\;\mathrm{for}\;\;\;\;f\leq 1\;MHz$$

Equation (8) relates to the left-pectoral ventricle implantation site, however, it does not take into account the effects of different implantation sites. Applied to our study this equation would mean a factor of approximately 50 between the voltage induced in unipolar and bipolar lead. In fact, for the different implantation sites investigated we derived factors between 1.3 and 7.6 as depicted in Table 4 (homogeneous exposure).

**Table 4:** Comparison of the factor ($u_{\text{uni}}/u_{\text{bi}}$) of the voltage induced in unipolar and bipolar leads at the four implantation sites.

Setup	LPV	LPA	RPV	RPA
Homogeneous exposure	5.8	7.6	1.8	1.3
Qi-A13-Board power transfer	9.6	15.0	7.4	7.1
Qi-A13-Board pinging (0 cm)	8.1	22.0	6.7	6.9

Mattei *et al*. [33] measured the voltage induced in unipolar and bipolar leads with a phantom model and coils of two different diameters (30 cm and 10 cm) for the exposure. They scrutinized magnetic fields with a frequency of 125 kHz and different implantation sites. The study design closely resembles the experimental setup of our study. Mattei *et al*. concluded that the voltage induced in a bipolar lead is reduced by a factor of 3–6, which lies within our findings (*cf.* Table 4). Additionally, Mattei *et al*. [33] presented that the voltage in a bipolar lead induced by using a 10 cm-coil reaches a maximum if it is positioned in a more curved path which complies with our findings that the induced voltage is higher at the atrium position for inhomogeneous fields.
